# Intraoperative change in distal motor latency as a predictor for clinical outcome after mini-OCTR: a retrospective cohort study

**DOI:** 10.3389/fneur.2025.1607199

**Published:** 2025-08-13

**Authors:** Mingjie Zhou, Chuxiang Chen, Chenpei Xu, Biao Yan, Philippe Liverneaux, Su Jiang

**Affiliations:** ^1^Department of Hand Surgery, Huashan Hospital, Fudan University, Shanghai, China; ^2^National Health Commission Key Laboratory of Hand Reconstruction, Shanghai, China; ^3^Shanghai Key Laboratory of Peripheral Nerve and Microsurgery, Shanghai, China; ^4^National Clinical Research Center for Aging and Medicine, Huashan Hospital, Fudan University, Shanghai, China; ^5^Institute of Hand Surgery, Shanghai, China; ^6^Institute of Hand Surgery, Fudan University, Shanghai, China; ^7^State Key Laboratory of Medical Neurobiology, MOE Frontiers Center for Brain Science and Institutes of Brain Science, Fudan University, Shanghai, China; ^8^Department of Hand Surgery, Strasbourg University Hospitals, Strasbourg, France; ^9^ICube CNRS UMR7357, Strasbourg University, Strasbourg, France; ^10^Department of Hand and Upper Extremity Surgery, Jing'an District Central Hospital, Shanghai, China

**Keywords:** carpal tunnel syndrome, distal motor latency, mini-OCTR, intraoperative electrophysiology, prognostic biomarker, sensory recovery

## Abstract

**Introduction:**

Although mini-open carpal tunnel release (Mini-OCTR) proves to be a standard solution for carpal tunnel syndrome (CTS), precise prediction of recovery remains challenging. The aim of this study was to explore the potential of using intraoperative change in distal motor latency (DML) to predict clinical outcomes.

**Methods:**

A retrospective cohort analysis was performed on 52 primary CTS patients, who completed the questionnaires before Mini-OCTR, 1 day (1 day Post-op) and 6 months after Mini-OCTR (6 months Post-op). Latency recovery percent (LRP) was calculated to represent intraoperative change of DML after Mini-OCTR. Multivariate and simple logistic regression analyses were used to quantify the predictive value of LRP on postoperative outcomes.

**Results:**

The results of patient-reported outcome measures (PROMs) demonstrated that Mini-OCTR was an effective procedure in treating CTS generally with some of the patients experiencing significant improvement in sensory function at 1 day Post-op. Multivariate logistic regression analysis which involves demographic information, CTS-related medical history, electrodiagnostic test results, PROMs and LRP revealed that the prognostic model has high AUC and accuracy, and LRP is a significant predictor among all the involved variables. Simple logistic regression analysis identified an optimal LRP cut-off value of 0.11 for predicting sensory recovery at 1 day Post-op with high accuracy.

**Conclusion:**

This study introduces LRP as a practical biomarker that enables surgeons to predict immediate postoperative sensory improvement in Mini-OCTR patients, which can assist surgeons in setting short-term expectations and tailoring postoperative care for the patients.

## Introduction

Carpal Tunnel Syndrome (CTS) is the most common nerve entrapment disorder to be diagnosed and treated globally, which is characterized by structural abnormalities at the compression site and impaired nerve conduction across the carpal tunnel (1, 2). The symptoms of CTS include tingling, numbness or pain at the distribution area of the median nerve in the hand with weakness of the thumb (3). Several risk factors contribute to its development, including genetics, metabolic disorders (such as diabetes, obesity), rheumatoid arthritis, acromegaly, hypothyroidism and hormonal fluctuations during pregnancy (4–8). The pathology of CTS is complicated (9), making it more difficult to predict the outcome (10–12). In detail, mild CTS is characterized by ischemia or a transient depolarization block which results in decreased conductivity of nerves (13, 14). Severe or prolonged compression will lead to focal complete demyelination in some nerve fibers and Wallerian degeneration with axonal loss in others, and a regenerative response in some neurons and related Schwann cells can also be observed at the same time (15, 16). Furthermore, pathological accumulation of amyloid fibrils in the carpal tunnel, often causing bilateral CTS, serves as an early sentinel sign of systemic amyloidosis (17, 18), and may precede the onset of cardiac amyloidosis by 5–10 years (19).

The current clinical approach to diagnosing CTS relies on a detailed medical history, physical examination, imaging techniques and electrodiagnostic (EDX) test (20, 21). Among these, nerve conduction studies (NCS) and electromyography (EMG) are crucial for diagnosing and grading CTS severity (20). These studies provide objective assessments of nerve function, with prolonged distal motor latency (DML) serving as a key indicator of nerve conduction damage (21). Based on the unique pathology of CTS, median nerve can exhibit immediate and long-term functional or structural changes after mini-open carpal tunnel release (Mini-OCTR), an effective and standardized procedure that can remove entrapment of median nerve achieve favorable outcomes in most cases (22–25). Although the time course of DML change has been reported (26–28), intraoperative (Intra-op) change in DML have been seldom researched, and their clinical value remains uncertain due to a lack of robust evidence linking these changes to postoperative (Post-op) outcomes (29, 30).

To clarify the clinical value of Intra-op changes in DML, we calculated latency recovery percent (LRP) based on preoperative (Pre-op) and Intra-op DML, and explore the prognostic value of LRP for predicting outcomes after Mini-OCTR. We hypothesized that a greater LRP would be associated with better outcomes and faster recovery after Mini-OCTR.

## Materials and methods

### Patient selection

This study included patients who received Mini-OCTR by our treatment group at the Hand Surgery Department of our hospital from February 2022 to May 2024. The diagnosis of CTS was made clinically and confirmed with nerve conduction studies. Patients met electrodiagnostic criteria for the diagnosis of CTS if DML across the carpal tunnel was over 4.5 msec. Exclusion criteria included a history of traumatic nerve injury, revision Mini-OCTR, coexisting neurological conditions (such as cubital tunnel syndrome or cervical spondylosis) and lack of Pre-op EMG and NCS reports from our hospital. CTS-related medical histories were documented for all participants. Histories of hypertension and diabetes were also recorded to investigate their potential impact on CTS prognosis.

**Figure 1:**
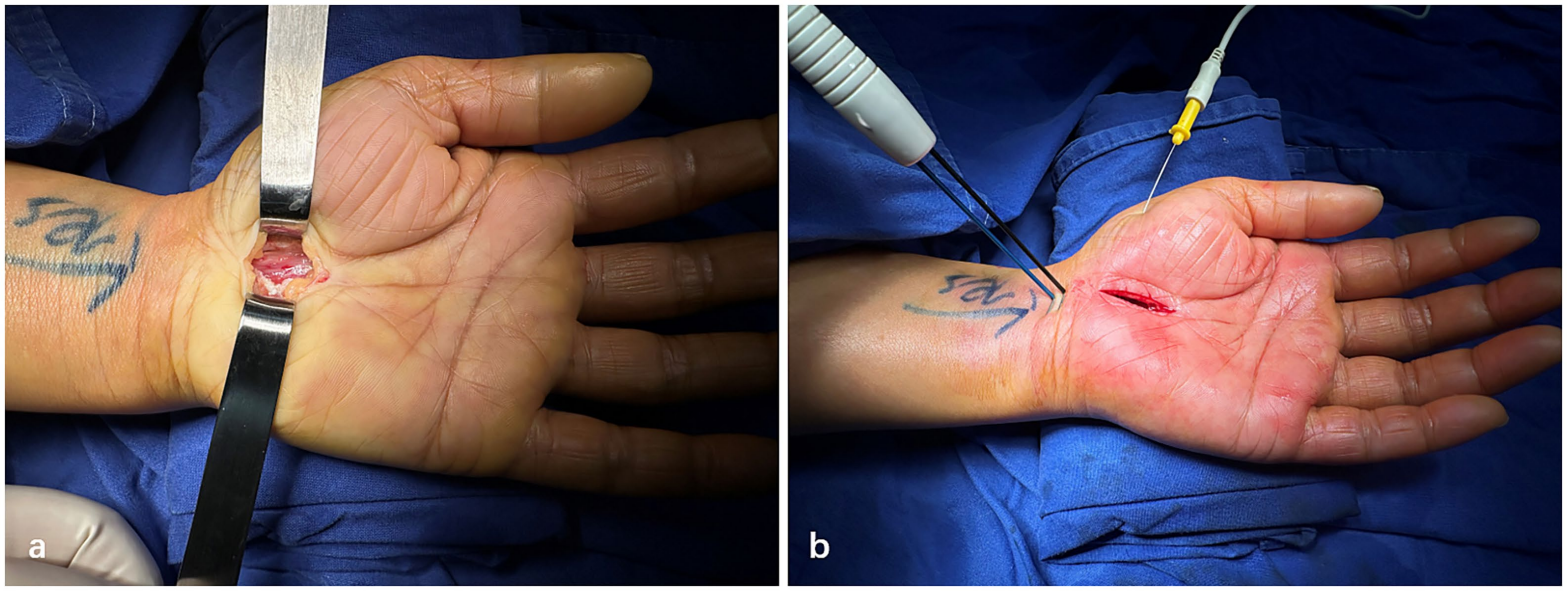
Representative photographs of mini-open carpal tunnel release and intraoperative electrophysiology test. **(a)** Intraoperative photographs after mini-Open carpal tunnel release. **(b)** Procedures of intraoperative electrophysiology test.

All patients underwent Mini-OCTR surgery under local anesthesia performed by the same surgeon at our hospital (Figure 1a). A tourniquet will be applied on the upper arm during the surgery. The technicians from the EMG department in our hospital performed the Pre-op and Intra-op EDX tests with standard operating procedure. A needle electrode was used over the abductor pollicis brevis muscle for recording, and the location of the stimulating electrode is at the proximal end of the wrist crease (Figure 1b). Intra-op electrical stimulation and electrophysiological test were conducted 5 min after Mini-OCTR and tourniquet release. The measurements were repeated at least five times until stable results were obtained. After Mini-OCTR, all the patients received standard care, including a 14-day cast immobilization, routine dressing changes and standardized hand rehabilitation involving nerve and tendon gliding exercises. They were prescribed oral methylcobalamin, vitamin B1, and vitamin B6 as neurotrophic agents, but no analgesics. Follow-up visits occurred 14 days postoperatively to monitor progress and remove sutures.

### Study design and outcomes

All the patients were asked to complete questionnaires before, 1 day after and 6 months after Mini-OCTR. The online questionnaires include Boston Carpal Tunnel Questionnaire (BCTQ) and Visual Analogue Scale (VAS). The BCTQ, consistent with previous studies, consists of two domains: Symptom Severity Scale (BCTQ-SSS) and Function Status Scale (BCTQ-FSS), comprising 11 and 8 items, respectively (31). Scores range from 1 to 5 (1 = no complaints, 5 = maximum complaints possible). VAS was used to measure pain from the distribution area of the median nerve in the hand (VAS-Pain) and hand function (VAS-Function) on a scale of 0 to 100 (0 = extreme pain/disability, 100 = no pain/disability), as well as patient satisfaction (VAS-satisfaction). Patients were categorized as satisfied or unsatisfied group using different criteria: (1) VAS score ≥ 60 vs. < 60; (2) BCTQ scores meeting the minimal clinically important difference (MCID), defined as > 0.8 for BCTQ-SSS, > 0.5 for BCTQ-FSS, or > 0.74 for BCTQ-total (32), as reported in the previous studies.

Demographic information, including age, gender, BMI, durations of diabetes and hypertension was recorded as potential predictors. CTS-related medical history including duration of CTS, smoking, occupation, dominate side of hand and thenar atrophy were also recorded. Several Pre-op EDX results were involved as well, including (1) EMG studies results including fibrillation potential, positive sharp wave, and recruitment phase of abductor pollicis brevis muscle; (2) DML of the median nerve to the abductor pollicis brevis muscle.

### Statistical analysis

Demographic information and CTS-related variables were described using frequencies (%) or means ± standard deviation. The results from PROMs were first tested for normal distribution with Kolmogorov–Smirnov test. Since the scores did not distribute normally, we subsequently conducted Friedman test followed by multiple comparisons (VAS-Pain, VAS-Function) or Wilcoxon matched-pairs signed rank test (BCTQ-SSS, BCTQ-FSS, BCTQ-Total).

Afterwards, we defined LRP to quantitatively the change between Pre-op and Intra-op DML. LRP was calculated as follows:


$$\mathrm{LRP}=\frac{{\mathrm{DML}}_{\mathrm{Pre}-\mathrm{op}}-{\mathrm{DML}}_{\text{Intra}-\mathrm{op}}}{{\mathrm{DML}}_{\mathrm{Pre}-\mathrm{op}}}$$

To explore the correlation between LRP and patient short-term (VAS-Pain-Post-op 1d and VAS-Function-Post-op 1d), long-term outcomes (VAS-Pain-6 months, VAS-Function-6 months, BCTQ-SSS-6 months, BCTQ-FSS-6 months, BCTQ-Total-6 months) and satisfaction (VAS-Satisfaction), we divided the patients into satisfied and unsatisfied groups according to these 8 scores and compared LRP between these two groups. The distribution of LRP in each group will first be tested for normality. Then either unpaired *t*-test or Mann–Whitney U test will be conducted based on the results of the normality test.

Categorical variables were converted into numerical variables in the logistic regression model (details shown in Supplementary Table S1). Firstly, multivariable logistic regression and Pearson correlation analysis were used to find the independent predictors of the outcomes after Mini-OCTR. Then, simple logistic regression was performed to determine the best cut-off value of LRP for prediction, which is identified by locating the point on the ROC curve where the trade-off between the true positive rate and the false positive rate is most favorable.

Sankey diagrams were generated using Origin 2022. Unpaired *t*-tests and plots were created using GraphPad Prism 9, while multivariable logistic regression, simple logistic regression, Pearson correlation analysis and ROC curves were conducted and plotted using Python scripts.

## Results

### Study cohort

**Figure 2:**
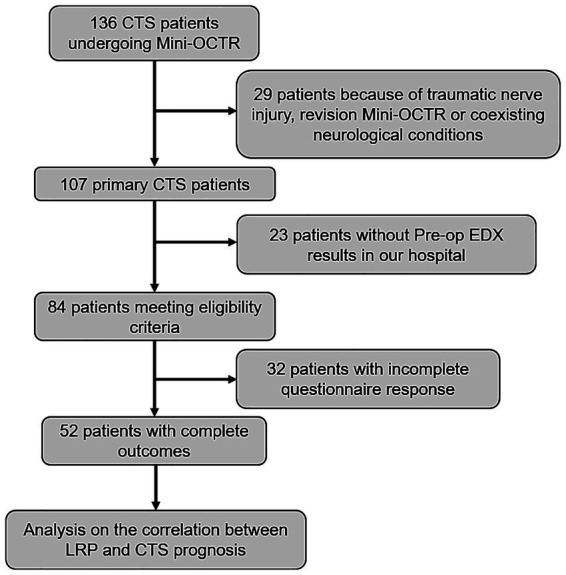
Study flowchart showing excluded patients. CTS, carpal tunnel syndrome. Mini-OCTR, mini-open carpal tunnel release. LRP, latency recovery percent.

A total of 136 patients underwent Mini-OCTR by our group. Among these, 29 cases were excluded due to a history of wrist trauma resulting in nerve damage, coexisting conditions affecting hand function (such as cubital tunnel syndrome or cervical spondylosis) or undergoing a revision Mini-OCTR at our hospital. Twenty three patients were excluded for lack of Pre-op EDX reports within 2 weeks before Mini-OCTR from the EMG department in our hospital. After applying eligibility criteria, 84 cases were included in the study. Among these cases, 52 patients had filled in all the questionnaires at Pre-op, 1 day Post-op and 6 months Post-op (Figure 2). And all the baseline characteristics of these 52 patients are presented in Table 1.

**Table 1:** Patient characteristics.

Variable	Patients (*n* = 52)
Age (year)	56.42(12.21)
Gender (%)	
Male	13(25%)
Female	39(75%)
Hand dominance (%)	
Left	3(6%)
Right	49(94%)
Affected side (%)	
Left	14 (29%)
Right	37(71%)
Hypertension (%)	
No	40(77%)
Yes	12(23%)
Diabetes mellitus (%)	
No	47(90%)
Yes	5(10%)
Smoking (%)	
Non-smoker	48(92%)
Former smoker	2(4%)
Current smoker	2(4%)
Duration of symptoms (mo)	14(23)
Body mass index	25.35(4.93)
Occupational intensity (%)	
Not employed	34(65%)
Light	6(12%)
Moderate	8(15%)
Severe	4(8%)
Thenar muscle atrophy (%)	
Yes	23(44%)
No	29(56%)

Data are given as the median (IQR) for continuous variables and as the frequency (percentage) for categorical variables. IQR, Inter Quartile Range.

### Surgical outcome

**Figure 3:**
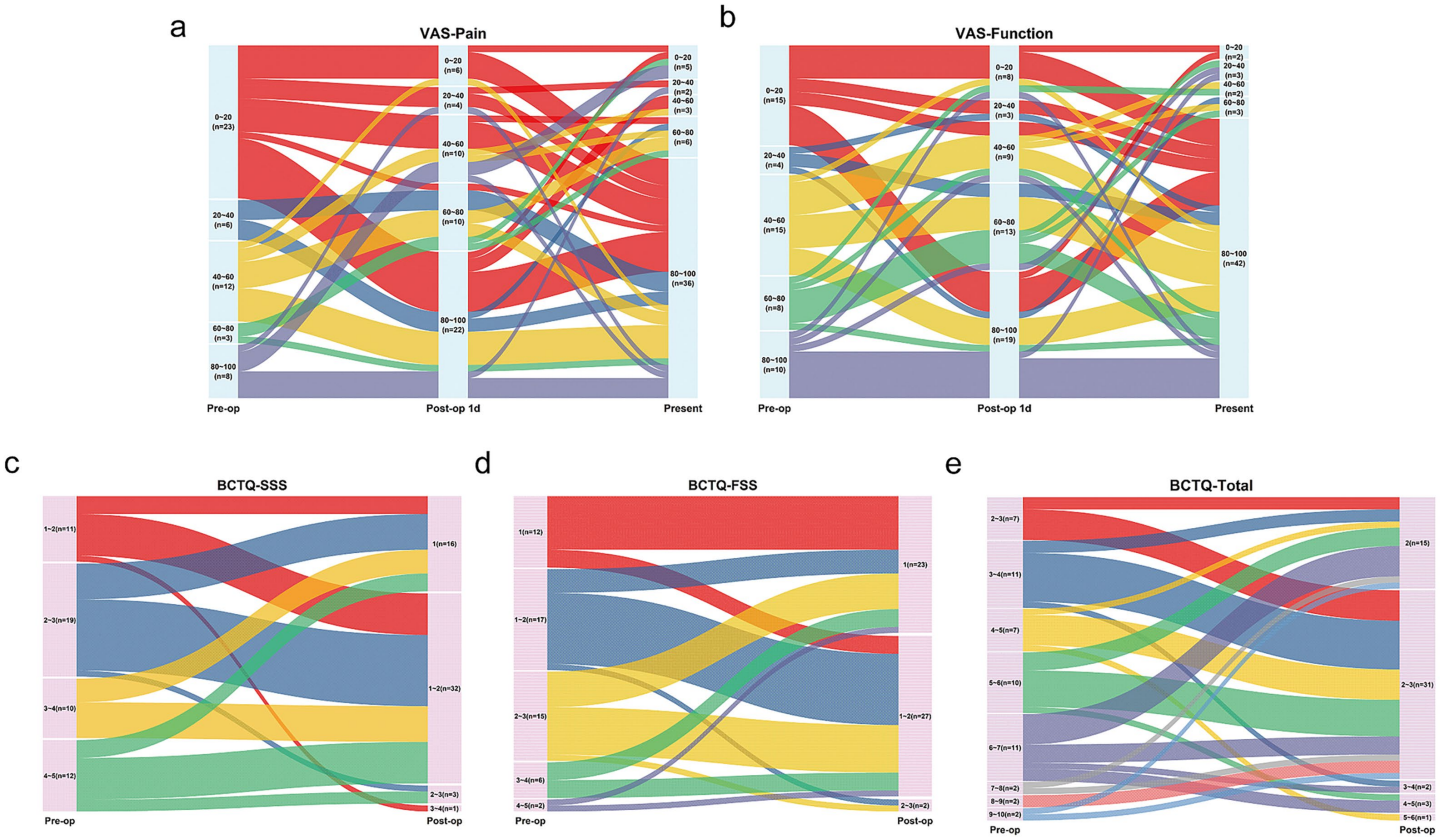
Changes in VAS and BCTQ scores after Mini-OCTR depicted by Sankey diagrams (*n* = 52). **(a,b)** The changes of VAS-Pain and VAS-function scores before (Pre-op, left), 1 day (middle) and 6 months (right) after Mini-OCTR. **(c–e)** The changes of BCTQ-SSS, BCTQ-FSS, BCTQ-Total scores before (Pre-op, left) and 6 months (right) after Mini-OCTR. VAS, visual analogue scale. BCTQ, boston carpal tunnel questionnaire.

Table 2 shows the median (interquartile range) of all primary and secondary outcomes for the 52 patients who completed all the follow-up questionnaires. The distribution of each score were illustrated in the Sankey diagrams (Figure 3), which showed the dynamic changes in these outcomes from Pre-op through 1 day Post-op to 6 months Post-op.

**Table 2:** Patient-reported outcome measurements.

Variable	Patients (*n* = 52)
VAS score	
VAS-pain (Pre-op)	20(50)
VAS-pain (1 day Post-op)	60(40)
VAS-pain (6 months Post-op)	90(30)
VAS-function (Pre-op)	50(60)
VAS-function (1 day Post-op)	60(31.25)
VAS-function (6 months Post-op)	90(20)
VAS-satisfaction (6 months Post-op)	100(20)
BCTQ-SSS	
BCTQ-SSS (Pre-op)	2.91(1.5)
BCTQ-SSS (6 months Post-op)	1.18(0.48)
BCTQ-FSS	
BCTQ-FSS (Pre-op)	1.94(1.5)
BCTQ-FSS (6 months Post-op)	1.13(0.25)
BCTQ-total	
BCTQ-Total (Pre-op)	2.54(1.37)
BCTQ-Total (6 months Post-op)	1.21(0.36)

Data are given as the median (IQR). IQR, Inter Quartile Range.

As for the BCTQ questionnaire scores, 49 out of 52 patients showed improvement in the total BCTQ score, with a median improvement of 2.22 points. Forty seven patients showed improvement in the BCTQ-SSS, with a median improvement of 1.73 points, and 33 patients showed improvement in the BCTQ-FSS, with a median improvement of 0.38 points. Improvements in the BCTQ-SSS (*p* < 0.0001), BCTQ-FSS (*p* < 0.0001), and BCTQ-Total (*p* < 0.0001) were statistically significant.

As for VAS-Pain and VAS-Function scores, 41 patients reported significant Pre-op tingling and pain (VAS-Pain-Pre-op<60), with 61.0% of these patients (25/41) experiencing noticeable improvement by 1 day Post-op (VAS-Pain-Post-op 1d ≥ 60). Overall, the median improvement in VAS-Pain-Post-op 1d was 30 points with statistical significance (adjusted *p* = 0.0012). 82.7% (43/52) patients reported improvement in hand tingling and pain 6 months Post-op, with a median improvement of 50 points with statistical significance (adjusted *p* < 0.0001). Regarding hand function, 9 patients reported decreased hand function at 1 day Post-op, but 2 of these reported improved function 6 months Post-op. Overall, 86.5% (45/52) patients reported improved hand function 6 months Post-op, with a median improvement of 45 points with statistical significance (adjusted *p* < 0.0001).

### Multivariable and simple logistic regression of LRP predicting surgery outcomes

**Figure 4:**
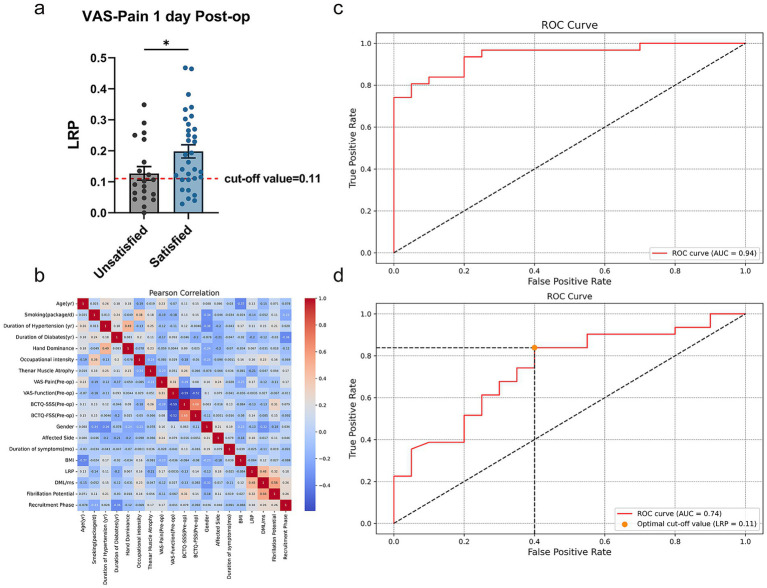
Results of multivariate and simple logistic regression. **(a)** Unpaired t test of LRP in unsatisfied and satisfied group divided by VAS-Pain 1 day Post-op. **(b)** Pearson correlation analysis of all potential predictors used in the multivariate logistic regression. **(c)** ROC curve of multivariate logistic regression of all the potential predictors for VAS-Pain 1 day Post-op. **(d)** ROC curve of simple logistic regression and optimal cut-off value of LRP for the prediction of VAS-Pain 1 day Post-op. ROC curve, receiver operating characteristic curve. *p < 0.05, unpaired *t* test. Data are presented as mean ± s.e.m.

As shown in Table 3, LRP differed significantly between the satisfied and unsatisfied groups only when classified by VAS-Pain-Post-op 1d (*p* = 0.0028). These results suggest that LRP is significantly associated with hand sensory recovery at 1 day Post-op, consistent with our clinical experience that greater LRP indicates better sensory recovery (Figure 4a).

**Table 3:** Comparison and statistical significance of LRP grouped by different standard.

Classification criteria	Unsatisfied group	Satisfied group	*P*-value
VAS-Pain (Pre-op)*	0.13(0.18), *n* = 41	0.19(0.17), *n* = 11	0.3895
VAS-Pain (1 day Post-op)#	0.10(0.13), *n* = 20	0.18(0.16), *n* = 32	0.0306*
VAS-Pain (6 months Post-op)*	0.18(0.14), *n* = 10	0.13(0.18), *n* = 42	0.5465
VAS-Function (Pre-op)*	0.13(0.19), *n* = 34	0.13(0.14), *n* = 18	0.6437
VAS-Function (1 day Post-op)*	0.12(0.19), *n* = 20	0.14(0.16), *n* = 32	0.5473
VAS-Function (6 months Post-op)*	0.19(0.08), *n* = 7	0.13(0.19), *n* = 45	0.6914
VAS-Satisfaction (6 months Post-op)*	0.08(0.10), *n* = 4	0.14(0.17), *n* = 48	0.3577
BCTQ-SSS (6 months Post-op)*	0.18(0.17), *n* = 12	0.13(0.18), *n* = 40	0.7529
BCTQ-FSS (6 months Post-op)*	0.13(0.21), *n* = 28	0.13(0.18), *n* = 24	0.9022
BCTQ-Total (6 months Post-op)#	0.12(0.21), *n* = 18	0.14(0.17), *n* = 34	0.7284

*Data are given as the median (IQR) and compared by Mann–Whitney U test. #Data are given as the mean (SD) and compared by unpaired *t*-test.

Afterwards, we included demographic information, Pre-op BCTQ and VAS scores, CTS-related variables, Pre-op EDX results and LRP in a multivariate logistic regression analysis. Figure 4b showed the values of Pearson Correlation Coefficient (PCC) between each predictor. Due to significant collinearity between BCTQ-SSS, BCTQ-FSS, BCTQ-Total and ipsi-F, ipsi-PS, we removed BCTQ-Total and ipsi-PS from subsequent analyses, where most absolute PCC values did not exceed 0.5, indicating no collinearity issues. ROC curve illustrated that this prognostic model has good discriminative ability with AUC = 0.94 (Figure 4c), Accuracy = 0.86, Precision = 0.87, Recall = 0.90, and F1 Score = 0.89. Among all the potential predictors involved in the multivariable logistic regression, LRP is the most significant independent predictor for sensory improvement at 1 day Post-op (*p* = 0.012, Odds Ratio = 37.922, *β* = 3.636 ± 1.453). (More details shown in Table 4) Subsequently, simple logistic regression analysis indicated that the optimal cut-off value of LRP for the prediction of sensory improvement at 1 day Post-op is 0.11, with an AUC = 0.74 (Figure 4d), Accuracy = 0.75, Precision = 0.76, Recall = 0.84, and F1 Score = 0.80. These results indicated that LRP can serve as a significant predictor for sensory improvement at 1 day Post-op with good predictive accuracy.

**Table 4:** Predicting sensory recovery at 1 day Post-op.

Predictors	β	SD	Odds ratio	*Z*-value	*P*-value
Age (yr)	0.815	0.876	2.258	0.929	0.353
Smoking (package/d)	2.851	1.309	17.306	2.178	0.029
Duration of hypertension (yr)	−0.661	0.863	0.517	−0.765	0.444
Duration of diabetes (yr)	0.090	0.763	1.094	0.117	0.907
Hand dominance	1.290	0.853	3.634	1.513	0.130
Occupational intensity	−1.343	0.837	0.261	−1.605	0.108
Thenar muscle atrophy	0.868	0.922	2.381	0.941	0.347
VAS-pain (Pre-op)	−0.698	0.682	0.498	−1.024	0.306
VAS-function (Pre-op)	0.046	0.785	1.047	0.059	0.953
BCTQ-SSS (Pre-op)	−3.275	1.617	0.038	−2.026	0.043
BCTQ-FSS (Pre-op)	0.455	1.189	1.576	0.382	0.702
Gender	3.034	1.462	20.786	2.076	0.038
Affected Side	0.835	0.687	2.304	1.215	0.224
Duration of symptoms (mo)	0.260	0.428	1.297	0.607	0.544
BMI	2.484	1.267	11.988	1.960	0.050
LRP	3.636	1.453	37.922	2.502	0.012*
DML (ms)	−0.423	0.905	0.655	−0.468	0.640
Fibrillation potential	−2.109	1.332	0.121	−1.583	0.113
Recruitment phase	2.007	1.092	7.438	1.838	0.066

The *p* value of LRP was marked with * to emphasize that it is an independent predictor.

## Discussion

Current clinical evidence suggests that sensory function typically improves earlier than motor function, with some severe CTS cases requiring up to 6 months for motor recovery postoperatively (33). In our clinical practice, we observed that some patients experienced obvious relief from numbness or pain 1 day after Mini-OCTR, whereas others did not show immediate improvement. This variability in recovery may confuse patients and affect their satisfaction with the surgery. We noted that this immediate improvement might correlate with LRP. Despite the fact that Intra-op electrophysiology technique during CTR has been reported for decades (29, 30). LRP is still a parameter seldom mentioned in the literature with its clinical value remaining to be explained. Thus, we conducted a follow-up study of CTS cases for 6 months and found a correlation between LRP and sensory improvement at 1 day Post-op. We emphasized the importance of the immediate improvement in sensory function after surgery, because it can notably improve the patient’s life quality, which has been confirmed in previous studies (34, 35). In our clinical practice, most patients are satisfied with the surgical outcome because of good sleep quality at night without hand discomfort at 1 day Post-op. Conversely, if the surgery does not resolve their sleep issues, which can be their primary concern, they may perceive the procedure as ineffective, leading to their dissatisfaction with the Post-op outcomes.

Our study demonstrated that LRP is an independent predictor of sensory improvement at 1 day Post-op, which shed light on the real-time, Intra-op utility of EDX tests for outcome prediction after Mini-OCTR. Critically, an LR *p* value below 0.11 serves as an objective Intra-op warning sign of a high likelihood of suboptimal sensory recovery within the first 24 h after surgery. This immediate predictive capability offers substantial clinical utility beyond prognostication.

Firstly, the availability of LRP offers a potential opportunity for Intra-op reassessment. A low LRP value (< 0.11), particularly encountered in cases of severe or chronic CTS, might prompt the surgeon to re-evaluate the completeness of the median nerve decompression under the mini-open incision. This could involve meticulous inspection for any residual constriction at the distal edge of the transected transverse carpal ligament, ensuring maximal nerve decompression. Furthermore, this finding raises important questions for surgical technique refinement. In response to low LRP values, we have modified our technique to attempt a more thorough decompression, sometimes utilizing a longer incision than the standard mini-open approach. Most importantly, LRP enables surgeons to set precise expectations: patients with LR *P* < 0.11 can be proactively counseled that while decompression is complete, significant sensory relief may evolve gradually over days or weeks, mitigating anxiety and preventing dissatisfaction from unmet immediate hopes. Furthermore, identifying these patients allows for optimized Post-op care, such as scheduling early proactive follow-up to reinforce recovery timelines and manage concerns, ensuring targeted support reaches those at higher risk for early disappointment.

Thus, LRP transforms an Intra-op measurement into a tool for enhancing patient-centered care. By enabling real-time identification of patients prone to delayed sensory recovery, LRP facilitates critical actions: potential Intra-op checks, personalized expectation management, and tailored early support. This directly addresses a key driver of early Post-op concern—immediate symptom relief—significantly improving the initial patient experience and satisfaction.

Several machine learning or deep learning models on ultrasound images or EDX test have been developed for CTS diagnosis and severity classification so far (36–40). And prognosis models for CTS outcomes have also been previously reported, focusing primarily on BCTQ-SSS scores or VAS scores, which have achieved predictions comparable to those made by professional hand surgeons (10–12, 41–43). Furthermore, increasing studies have created models combined clinical data with histological and imaging results (44–47). These multi-classifier systems integrated by clinico-histology-genomic analysis makes clinical predictions more accurate and effective. Although such models often focus on the diagnosis and prognosis of diseases like cancer, this is also a direction for the development of CTS prognosis models. Given the current tendency that the importance of EDX tests is gradually being complemented by imaging studies (48), such model can become a comprehensive tool for clinical diagnosis and prognosis prediction.

While our analysis focused on demographic and electrodiagnostic predictors, it is important to acknowledge other perioperative variables that may contribute to early sensory outcomes. For instance, Post-op analgesic regimens such as gabapentinoids have been shown to significantly improve nocturnal symptom severity and sleep quality in patients with residual symptoms after CTR, though they do not affect functional status or daytime numbness (49). Similarly, adherence to Post-op immobilization (50) and specialized physical therapy (51) are other factors that may affect early axonal microenvironment recovery. Regarding surgical techniques, recent research has confirmed that the wide awake local anesthesia no tourniquet (WALANT) technique and local anesthetic with a tourniquet (LA-T) yield similar results in Post-op pain, suggesting that tourniquet time may not be a key determinant of pain (52). However, in the context of nerve function itself, tourniquet time can have a very significant impact due to ischemia, despite the similarity in Post-op pain outcomes. As these factors were standardized as part of our protocol, they were not specifically analyzed. We propose that future studies can systematically document surgical technical details, analgesic use patterns, and rehabilitation compliance. This would better contextualize LRP’s predictive role.

The most important limitation in our study was the small sample size. Next, our follow-up only captured the sensory improvement at 1 day and 6 months Post-op, limiting our exploration of the relationship between LRP and the time course of hand sensory and function recovery. Our results indicated no obvious correlation between LRP and recovery at the final follow-up point (6 months). Nevertheless, based on our 52-case cohort, sensory recovery at 1 day Post-op did not regress over the subsequent 6 months, suggesting that patients with high LRP might experience faster sensory recovery, but the difference of function recovery may diminish when the final follow-up time points is 6 months. Despite that short-term change in DML after CTR have been confirmed unconcerned with the long term outcomes in some studies (26), we believe the predictive value of LRP for long-term milestones such as earlier return to work or daily functional independence can be discovered with more follow-up time points and a larger cohort.

## Data Availability

The original contributions presented in the study are included in the article/supplementary material, further inquiries can be directed to the corresponding author.

## References

[1] Malakootian M Soveizi M Gholipour A Oveisee M. Pathophysiology, diagnosis, treatment, and genetics of carpal tunnel syndrome: a review. Cell Mol Neurobiol. (2023) 43:1817–31. doi: 10.1007/s10571-022-01297-2 36217059 PMC11412174

[2] Olney RK. Carpal tunnel syndrome: complex issues with a “simple” condition. Neurology. (2001) 56:1431–2. doi: 10.1212/wnl.56.11.1431 11402097

[3] Phalen GS. The carpal-tunnel syndrome. Seventeen years’ experience in diagnosis and treatment of six hundred fifty-four hands. J Bone Joint Surg Am. (1966) 48:211–28. doi: 10.2106/00004623-196648020-00001 5934271

[4] Sandy-Hindmarch O Bennett DL Wiberg A Furniss D Baskozos G Schmid AB. Systemic inflammatory markers in neuropathic pain, nerve injury, and recovery. Pain. (2022) 163:526–37. doi: 10.1097/j.pain.0000000000002386 34224495 PMC7612369

[5] Vernick RC Beckwitt CH Fowler JR. Subjective and objective differences in patients with unilateral and bilateral carpal tunnel syndrome and the role of obesity in syndrome severity. Plast Reconstr Surg. (2024) 153:423–9. doi: 10.1097/PRS.0000000000010773 37257136

[6] Lee K-H Lee C-H Lee B-G Park J-S Choi W-S. The incidence of carpal tunnel syndrome in patients with rheumatoid arthritis. Int J Rheum Dis. (2015) 18:52–7. doi: 10.1111/1756-185X.12445 25196946

[7] Pourmemari MH Shiri R. Diabetes as a risk factor for carpal tunnel syndrome: a systematic review and meta-analysis. Diabet Med. (2016) 33:10–6. doi: 10.1111/dme.12855 26173490

[8] Nyrhi L Kuitunen I Ponkilainen V Jokihaara J Huttunen TT Mattila VM. Incidence of peripheral nerve decompression surgery during pregnancy and the first year after delivery in Finland from 1999 to 2017: a retrospective register-based cohort study. J Hand Surg Am. (2023) 48:452–9. doi: 10.1016/j.jhsa.2023.01.013 36922291

[9] Dahlin LB Zimmerman M Calcagni M Hundepool CA Van Alfen N Chung KC. Carpal tunnel syndrome. Nat Rev Dis Primers. (2024) 10:37. doi: 10.1038/s41572-024-00521-1 38782929

[10] Hoogendam L Bakx JAC Souer JS Slijper HP Andrinopoulou E-R Selles RW. Predicting clinically relevant patient-reported symptom improvement after carpal tunnel release: a machine learning approach. Neurosurgery. (2022) 90:106–13. doi: 10.1227/NEU.0000000000001749 34982877

[11] Elseddik M Mostafa RR Elashry A El-Rashidy N El-Sappagh S Elgamal S. Predicting CTS diagnosis and prognosis based on machine learning techniques. Diagnostics. (2023) 13:492. doi: 10.3390/diagnostics13030492 36766597 PMC9914125

[12] Rezaee M Roshandel H Rahimibarghani S Rihani TSS Mohammadyahya E. Predictors of pain intensity in carpal tunnel syndrome: development and validation of a model. Clin Neurol Neurosurg. (2024) 243:108395. doi: 10.1016/j.clineuro.2024.108395 38936177

[13] Schmid AB Fundaun J Tampin B. Entrapment neuropathies: a contemporary approach to pathophysiology, clinical assessment, and management. Pain Rep. (2020) 5:e829. doi: 10.1097/PR9.0000000000000829 32766466 PMC7382548

[14] Nodera H Kaji R. Nerve excitability testing and its clinical application to neuromuscular diseases. Clin Neurophysiol. (2006) 117:1902–16. doi: 10.1016/j.clinph.2006.01.018 16631406

[15] Mackinnon SE Dellon AL Hudson AR Hunter DA. Chronic human nerve compression--a histological assessment. Neuropathol Appl Neurobiol. (1986) 12:547–65. doi: 10.1111/j.1365-2990.1986.tb00159.x 3561691

[16] Mackinnon SE. Pathophysiology of nerve compression. Hand Clin. (2002) 18:231–41. doi: 10.1016/s0749-0712(01)00012-9 12371026

[17] Donnelly JP Hanna M Sperry BW Seitz WH. Carpal tunnel syndrome: a potential early, red-flag sign of amyloidosis. J Hand Surg Am. (2019) 44:868–76. doi: 10.1016/j.jhsa.2019.06.016 31400950

[18] Lauppe RE Liseth Hansen J Gerdesköld C Rozenbaum MH Strand AM Vakevainen M. Nationwide prevalence and characteristics of transthyretin amyloid cardiomyopathy in Sweden. Open Heart. (2021) 8:e001755. doi: 10.1136/openhrt-2021-001755 34645699 PMC8515473

[19] Middleton SD Anakwe RE. Carpal tunnel syndrome. BMJ. (2014) 349:g6437. doi: 10.1136/bmj.g6437 25378457

[20] Osiak K Mazurek A Pękala P Koziej M Walocha JA Pasternak A. Electrodiagnostic studies in the surgical treatment of carpal tunnel syndrome-a systematic review. J Clin Med. (2021) 10:2691. doi: 10.3390/jcm10122691 34207345 PMC8235020

[21] Werner RA Andary M. Electrodiagnostic evaluation of carpal tunnel syndrome. Muscle Nerve. (2011) 44:597–607. doi: 10.1002/mus.22208 21922474

[22] Marti C Hensler S Herren DB Niedermann K Marks M. Measurement properties of the EuroQoL EQ-5D-5L to assess quality of life in patients undergoing carpal tunnel release. J Hand Surg Eur Vol. (2016) 41:957–62. doi: 10.1177/1753193416659404 27435748

[23] Miller LE Hammert WC Chung KC. Best-evidence systematic review and Meta-analysis of endoscopic carpal tunnel release outcomes. J Hand Surg Glob Online. (2023) 5:768–73. doi: 10.1016/j.jhsg.2023.07.011 38106929 PMC10721515

[24] Cagle PJ Reams M Agel J Bohn D. An outcomes protocol for carpal tunnel release: a comparison of outcomes in patients with and without medical comorbidities. J Hand Surg Am. (2014) 39:2175–80. doi: 10.1016/j.jhsa.2014.07.017 25218142

[25] Gerritsen AAM de Krom MCTFM Struijs MA Scholten RJPM de Vet HCW Bouter LM. Conservative treatment options for carpal tunnel syndrome: a systematic review of randomised controlled trials. J Neurol. (2002) 249:272–80. doi: 10.1007/s004150200004 11993525

[26] Rotman MB Enkvetchakul BV Megerian JT Gozani SN. Time course and predictors of median nerve conduction after carpal tunnel release. J Hand Surg Am. (2004) 29:367–72. doi: 10.1016/j.jhsa.2004.01.011 15140473

[27] Ginanneschi F Milani P Reale F Rossi A. Short-term electrophysiological conduction change in median nerve fibres after carpal tunnel release. Clin Neurol Neurosurg. (2008) 110:1025–30. doi: 10.1016/j.clineuro.2008.07.006 18845386

[28] El-Hajj T Tohme R Sawaya R. Changes in electrophysiological parameters after surgery for the carpal tunnel syndrome. J Clin Neurophysiol. (2010) 27:224–6. doi: 10.1097/WNP.0b013e3181dd4ff0 20461017

[29] Congiusta DV Yeranosian M Amer KM Abdelshahed D Yonclas P DalCortivo RL. Intraoperative nerve conduction studies during open carpal tunnel release: a pilot study. Eplasty. (2022) 22:e63. 36545639 PMC9748840

[30] Eversmann WW Ritsick JA. Intraoperative changes in motor nerve conduction latency in carpal tunnel syndrome. J Hand Surg Am. (1978) 3:77–81. doi: 10.1016/s0363-5023(78)80119-1 621369

[31] Atroshi I Johnsson R Sprinchorn A. Self-administered outcome instrument in carpal tunnel syndrome: reliability, validity and responsiveness evaluated in 102 patients. Acta Orthop Scand. (1998) 69:82–8. doi: 10.3109/17453679809002363 9524525

[32] Leite JCDC Jerosch-Herold C Song F. A systematic review of the psychometric properties of the Boston carpal tunnel questionnaire. BMC Musculoskelet Disord. (2006) 7:78. doi: 10.1186/1471-2474-7-78 17054773 PMC1624826

[33] Jansen MC Evers S Slijper HP De Haas KP Smit X Hovius SE. Predicting clinical outcome after surgical treatment in patients with carpal tunnel syndrome. J Hand Surg Am. (2018) 43:1098–1106.e1. doi: 10.1016/j.jhsa.2018.05.017 29945840

[34] Tulipan JE Kim N Abboudi J Jones C Liss F Kirkpatrick W. Prospective evaluation of sleep improvement following carpal tunnel release surgery. J Hand Surg Am. (2017) 42:390.e1–6. doi: 10.1016/j.jhsa.2017.02.009 28359641

[35] Atroshi I Gummesson C Johnsson R Sprinchorn A. Symptoms, disability, and quality of life in patients with carpal tunnel syndrome. J Hand Surg Am. (1999) 24:398–404. doi: 10.1016/s0363-5023(99)70014-6 10194028

[36] Tsukamoto K Matsui R Sugiura Y Fujita K. Diagnosis of carpal tunnel syndrome using a 10-s grip-and-release test with video and machine learning analysis. J Hand Surg Eur Vol. (2024) 49:634–6. doi: 10.1177/17531934231214661 37994011

[37] Elseddik M Alnowaiser K Mostafa RR Elashry A El-Rashidy N Elgamal S. Deep learning-based approaches for enhanced diagnosis and comprehensive understanding of carpal tunnel syndrome. Diagnostics. (2023) 13:3211. doi: 10.3390/diagnostics13203211 37892032 PMC10606231

[38] Bakalis D Kontogiannis P Ntais E Simos YV Tsamis KI Manis G. Carpal tunnel syndrome automated diagnosis: a motor vs. sensory nerve conduction-based approach. Bioengineering. (2024) 11:175. doi: 10.3390/bioengineering11020175 38391661 PMC10886232

[39] Tsamis KI Kontogiannis P Gourgiotis I Ntabos S Sarmas I Manis G. Automatic electrodiagnosis of carpal tunnel syndrome using machine learning. Bioengineering. (2021) 8:181. doi: 10.3390/bioengineering8110181 34821747 PMC8615235

[40] Park D Kim BH Lee S-E Kim DY Kim M Kwon HD. Machine learning-based approach for disease severity classification of carpal tunnel syndrome. Sci Rep. (2021) 11:17464. doi: 10.1038/s41598-021-97043-7 34465860 PMC8408248

[41] Loos NL Hoogendam L Souer JS van Uchelen JH Slijper HP Wouters RM. Algorithm versus expert: machine learning versus surgeon-predicted symptom improvement after carpal tunnel release. Neurosurgery. (2024) 95:110–7. doi: 10.1227/neu.0000000000002848 38299861 PMC11155572

[42] Bowman A Rudolfer S Weller P Bland JDP. A prognostic model for the patient-reported outcome of surgical treatment of carpal tunnel syndrome. Muscle Nerve. (2018) 58:784–9. doi: 10.1002/mus.26297 29981160

[43] Yetiş M Kocaman H Canlı M Yıldırım H Yetiş A Ceylan İ. Carpal tunnel syndrome prediction with machine learning algorithms using anthropometric and strength-based measurement. PLoS One. (2024) 19:e0300044. doi: 10.1371/journal.pone.0300044 38630703 PMC11023568

[44] Ström P Kartasalo K Olsson H Solorzano L Delahunt B Berney DM. Artificial intelligence for diagnosis and grading of prostate cancer in biopsies: a population-based, diagnostic study. Lancet Oncol. (2020) 21:222–32. doi: 10.1016/S1470-2045(19)30738-7 31926806

[45] Huang K-B Gui C-P Xu Y-Z Li X-S Zhao H-W Cao J-Z. A multi-classifier system integrated by clinico-histology-genomic analysis for predicting recurrence of papillary renal cell carcinoma. Nat Commun. (2024) 15:6215. doi: 10.1038/s41467-024-50369-y 39043664 PMC11266571

[46] Lu MY Chen TY Williamson DFK Zhao M Shady M Lipkova J. AI-based pathology predicts origins for cancers of unknown primary. Nature. (2021) 594:106–10. doi: 10.1038/s41586-021-03512-4 33953404

[47] Coudray N Ocampo PS Sakellaropoulos T Narula N Snuderl M Fenyö D. Classification and mutation prediction from non-small cell lung cancer histopathology images using deep learning. Nat Med. (2018) 24:1559–67. doi: 10.1038/s41591-018-0177-5 30224757 PMC9847512

[48] Inui A Nishimoto H Mifune Y Kokubu T Sakata R Kurosaka M. Ultrasound measurement of median nerve cross-sectional area at the inlet and outlet of carpal tunnel after carpal tunnel release compared to electrodiagnostic findings. Arch Orthop Trauma Surg. (2016) 136:1325–30. doi: 10.1007/s00402-016-2514-9 27481365

[49] Aydin M Argun G Acar B Arikan M Toğral G Cinaroglu S. Residual symptoms after carpal tunnel decompression and treatment with gabapentin: a multicenter study. Cureus. (2021) 13:e17638. doi: 10.7759/cureus.17638 34646686 PMC8485700

[50] Zhang F Jiang H Lu Z Yang H Zhang Q Mi J. The significance of wrist immobilization for endoscopic carpal tunnel release. Front Neurol. (2023) 14:1081440. doi: 10.3389/fneur.2023.1081440 37181552 PMC10167297

[51] Georgiew F Florek J Bębenek A Sobanski G Florek P Petrovych O. Assessment of the impact of specialized physical therapy on the clinical condition of patients after carpal tunnel release. Cureus. (2025) 17:e82529. doi: 10.7759/cureus.82529 40385812 PMC12085949

[52] Gallucci GL Rosa YC Cerrutti WG Tanoira I Rellán I. WALANT technique versus local anesthesia with a tourniquet in carpal tunnel syndrome. Arch Bone Jt Surg. (2023) 11:321–5. doi: 10.22038/ABJS.2023.62995.3052 37265528 PMC10231921

